# Intersectoral care management for older people with cognitive impairment during and after hospital stays [intersec-CM]: study protocol for a process evaluation within a randomised controlled trial

**DOI:** 10.1186/s13063-021-05021-1

**Published:** 2021-01-21

**Authors:** Terese Dehl, Ulf Sauerbrey, Adina Dreier-Wolfgramm, Angela Nikelski, Nino Chikhradze, Armin Keller, Jessica Laufer, Fanny Schumacher-Schoenert, Stefan Kreisel, Jochen René Thyrian, Wolfgang Hoffmann, Horst Christian Vollmar

**Affiliations:** 1 Institute of Community Medicine, Department of Epidemiology and Community Health, University Medicine Greifswald, Ellernholzstr. 1-2, 17489 Greifswald, Germany; 2 Institute of General Practice and Family Medicine, University Hospital Jena, Friedrich Schiller University Jena, Bachstr. 18, 07743 Jena, Germany; 3 Department of Social Work and Education, Neubrandenburg University of Applied Sciences, Neubrandenburg, Germany; 4 Department for Nursing Care and Management, Faculty of Business & Social Sciences, Hamburg University of Applied Sciences, Berliner Tor 5, 20099 Hamburg, Germany; 5 Division of Geriatric Psychiatry, Evangelisches Klinikum Bethel, Bethesdaweg 12, 33617 Bielefeld, Germany; 6 Institute of General Practice and Family Medicine (AM RUB), Medical Faculty, Ruhr University Bochum (RUB), Universitätsstr. 150, 44801 Bochum, Germany; 7 German Centre for Neurodegenerative Diseases (DZNE), site Rostock/ Greifswald, Ellernholzstr. 1-2, 17489 Greifswald, Germany

**Keywords:** Process evaluation, Implementation fidelity, Cognitive impairment, Dementia, Hospital discharge, Transition, Intersectoral care management, Case management, Complex intervention

## Abstract

**Background:**

In the healthcare system in Germany, different institutions and actors play specific roles in the discharge and transition of patients from hospitals into primary care (Sachverständigenrat zur Begutachtung der Entwicklung im Gesundheitswesen, Wettbewerb an der Schnittstelle zwischen ambulanter und stationärer Gesundheitsversorgung, 2012). However, there are shortcomings in these intersectoral transitions. Especially in older people with cognitive impairment (PCI), discharge management often lacks coordination and cooperation between healthcare providers. This frequently results in higher rates of unscheduled readmission. The project intersec-CM is a randomised controlled trial (RCT) that aims to explore up to what extent an intersectoral care management (ICM) can improve this transition. This ICM is delivered by nurses with special training in care management. The objective of this paper is to describe a mixed-methods process evaluation of the intersectoral care management intervention and the factors that facilitate and inhibit its implementation.

**Methods:**

Different study designs for process evaluations from previous literature were collected and analysed according to the dimension implementation fidelity, satisfaction with the intervention, feasible transfer into routine care, optimum point of time, frequency and execution of the intervention, and context factors.

**Results:**

The actor-network theory was chosen as the theoretic framework for the process evaluation. Based on this theory, a mixed-methods design was developed to combine and integrate qualitative and quantitative evaluation methods. The qualitative part includes semi-structured interviews using topic guides (phase 1) and later in-depth interviews with narrative portions (phase 3), which will be analysed by using the qualitative content analysis according to Kuckartz. The quantitative survey (phase 2) is conducted with standardised questionnaires.

**Discussion:**

Challenges in data collection include the development of interview guidelines, which require different terminologies depending on every specific actor targeted in the intervention. Conducting the interviews, there is a risk of misunderstanding the older PCI by the interviewer and vice versa. However, the combination of qualitative and quantitative approaches as different techniques of process evaluation may help to capture, integrate and analyse data on different dimensions of the intervention.

**Conclusions:**

The results of our process evaluation may serve as an implementation guideline for intersectoral care management in the German healthcare system. Furthermore, the approach to evaluate the process of a complex intervention in health care for older PCI may serve as a stimulus to broaden the evidence base also of other complex intervention studies to improve health care for this vulnerable group.

The study was ethically approved by the Ethics Committee of the Ernst-Moritz-Arndt University of Greifswald. The study has been registered at the U.S. National Library of Medicine.

**Trial registration:**

ClinicalTrials.gov NCT03359408. Registered on 2 December 2017. The approximate date when recruitment to the process evaluation of the study will be completed is 31 May 2021.

## Background

In Germany, care for older people with cognitive impairment (PCI) or dementia is provided by a strongly sectorised healthcare system offering outpatient treatment and care, inpatient treatment and care, and rehabilitation. Different institutions and actors are involved in these sectors [1]. Each actor plays a specific role in the discharge of patients from the hospital and the transition back to his/her home. However, there are shortcomings in such intersectoral transitions between institutions and in the cooperation of the various healthcare actors involved, rendering discharge management of the older people a challenge. It is known that people with dementia have higher rates of unscheduled readmission into the hospital in a 30-day follow-up, especially for patients who were discharged into their homes [2]. Caregiver reported a lack of consultation before hospital discharge, and furthermore, the discharge is sometimes unexpected and continuity of medical care and social support is limited [2–4]. As one example, older multimorbid patients often experience negative side effects by the simultaneous intake of a large number of different drugs [2–4]. This leads to insufficient treatment and care of the older, cost-intensive preventable re-hospitalisations and premature institutionalisation, and thus, to dissatisfaction of patients, caregivers and healthcare providers. These deficits refer to the transition between the different sectors and to the collaboration of the different actors and healthcare professionals, e.g. physicians, nurses and therapists. The intersec-CM study is an attempt to improve the situation by implementing an intersectoral care management (ICM) coordinated by specially trained nurses and to prepare an implementation guideline for routine care in the German healthcare system.

### The intervention programme in intersec-CM [5]

The conceptional framework for the intervention is the evidence-based Dementia Care Management that was positively evaluated in the DelpHi-MV trial [6]. The DelpHi-Care Management comprises a large number of different modules to optimise the care of people with dementia and their caregivers in the ambulatory setting. The DelpHi intervention was refined to a discharge management and is presently evaluated in the randomised controlled trial (RCT) intersec-CM [7]. At two study centres in Bielefeld and Greifswald, Germany, specially trained study nurses monitor the transition of the PCI from an acute care hospital back to their homes. In the intervention group, the nurses will conduct the discharge management. The intersec-CM intervention consists of [5]:

- A comprehensive assessment of the health and social status of each patient at the time of admission to the acute care hospital
- A comprehensive needs assessment at the time of admission to the hospital
- An assessment of caregiver health and burden
- Provision of a systematic, written feedback to the treating hospital staff as well as the receiving physicians and respectively the patients and their caregivers with specific recommendations for treatment and care after discharge (hospital information letter)

**Fig. 1:**
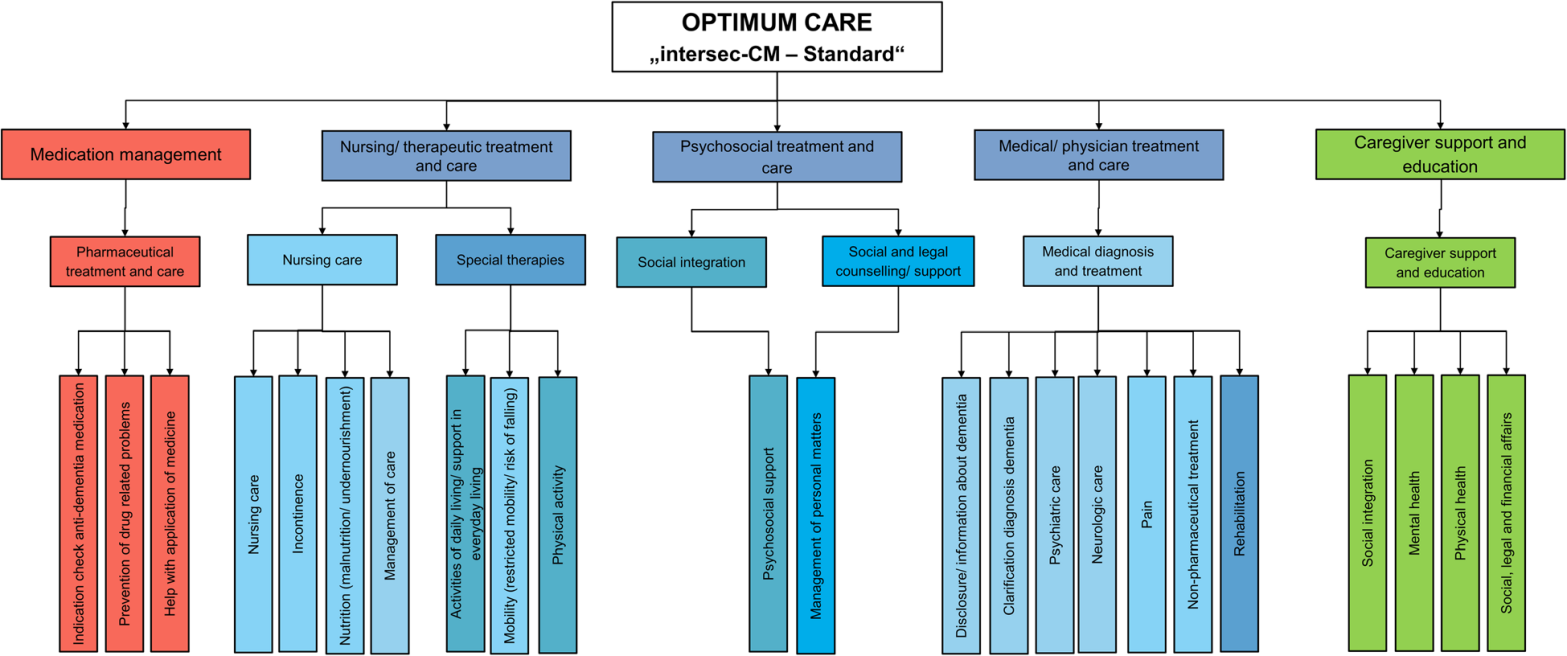
Optimum care as “DelpHi-standard” in the discharge management: pillars and action fields

The comprehensive needs assessment is based on the DelpHi-standard of optimum care [8]. Its adaption for the intersec-CM discharge management is illustrated in Fig. 1.

In this intersectoral care management, the study nurses are responsible for the assessment as well as the delivery of the individualised intervention. They develop, implement and monitor treatment and care, based on the assessments of patients’ and caregivers’ unmet needs [5]. This will be realised in close cooperation with the treating hospital, the PCI’s general practitioner (GP) and other ambulatory healthcare providers including home care services, occupational therapy, physiotherapy and speech therapy [5]. The selection of modules for the intervention will be supported by a tablet-computer-based Intervention-Management-System [9]. Specifically, this software supports the identification of each PCI’s and caregivers’ unmet needs and selects corresponding interventions for a PCI-specific individualised intervention plan [9]. The DelpHi trial standard and the computer system have proven of their functionality, acceptance and efficacy to improve health care and health-related endpoints of persons with dementia and their caregivers [8].

### Process evaluation frameworks

Alongside the intervention arm of the intersec-CM trial, a mixed-methods process evaluation is conducted to analyse which influencing factors facilitate and which inhibit its implementation. After a literature review, we analysed the existing frameworks for process evaluation. An often used framework by Baranowski and Stables is based on a study for cancer prevention [10]. Linnan and Steckler used this framework for public health interventions and established seven key dimensions of process evaluation: context (environmental aspects of the intervention setting), reach (the proportion of participants who received the intervention), fidelity (whether the intervention is delivered as planned), dose delivered and received (the amount of intervention delivered and the extent to which participants responded to it), implementation (a composite score of reach, dose and fidelity) and recruitment (methods used to attract participants) [11]. The UK Medical Research Council guidance for developing and evaluating complex interventions recommends to focus on differences between expected and observed outcomes. According to the Medical Research Council, “a mixture of qualitative and quantitative methods is likely to be needed” for process evaluations [10]. Furthermore, evaluation should help to understand how context influences outcomes and to provide insights to the implementation process [12].

Existing frameworks of process evaluation studies in dementia address similar dimensions. The deal-id study investigated how feedback for caregivers/relatives of persons with dementia elicits positive emotions. The corresponding process evaluation analysed sampling quality (recruitment and randomisation, reach) and intervention quality (relevance and feasibility, adherence to protocol) [13]. A process evaluation for dementia care mapping in Germany used adherence, dosage, participant responsiveness and quality of delivery as evaluation criteria [14]. The FallDem study evaluated the use of two different types of case conferences via delivery of intervention, response to intervention, recruitment and intervention context [15]. In a study of Prick and colleagues, where persons with dementia did exercises and got support, three dimensions were analysed: the success rate of recruitment and the quality of the *study population*, the quality of the complex *intervention* itself and the process of *data acquisition* in the study [16]. Sequential methods have to be used longitudinally over several data points [17]. Grant et al. stated that the use of qualitative methods in process evaluations is common [11, 18, 19].

## Methods

Intersec-CM implements and evaluates a complex intervention, which itself is affected by a set of contextual factors including the conditions of hospital discharge in the German heathcare system [20]. Trials investigating this double complexity are rare [21, 22]. To address the complexity and to discover the different components within the intervention (“open the black box”), we will use a multiphase mixed-methods design (three phases: qualitative, quantitative, qualitative). Our intention is to create insights into different dimensions of the intervention as well as to identify supporting and hindering aspects of the implementation according to different groups of actors in health care [23].

The design of our process evaluation is based on the study conducted by Prick et al. [16]. The dimensions of the study population and the data acquisition will be described by the research team of the intersec-CM trial [5]. The process evaluation in intersec-CM is focusing on the intervention itself. Based on further process evaluation designs [10–16], we defined dimensions and research questions focusing on relevant key actors of the ICM in routine care.

### Relevant actors of the ICM

To conduct the process evaluation of the ICM by the specially trained study nurses in a routine care setting, it is necessary to assess and examine the experiences of the key actors in healthcare provision for older PCI. At the participating clinics of the two recruiting study centres in intersec-CM, these actors include nurses, social workers, hospital physicians, general practitioners, study nurses and PCI and their caregivers/relatives. The choice to focus on these groups of actors, which form a more or less strong network in health care of older PCI, is based on the actor-network theory (ANT) by the sociologist Bruno Latour [24]. The ANT is a social science theory used to describe the composition of professional and private practices in modern societies and their institutions. We use this theory as a “theoretical lens” [24] to understand the social environment in which the actual intervention takes place [25]. As the healthcare system in Germany is based on different institutions and multiple actors, the ANT seems to be useful for our process evaluation, because in ANT’s centre there are not exclusively individual subjects and their actions, nor exclusively social structures that determine the actions of the subjects, but rather the associations between actors and networks. From ANT’s point of view, every participant in a network is related to a number of other actors that he/she cannot completely control, but to whom he/she owes his/her ability to act and with whom he/she shares his/her agency [26]. Against this background, the intersec-CM intervention comprises multiple interacting components and different organisational levels of health care at the discharge of older PCI from the hospital to the home.

## Results

**Fig. 2:**
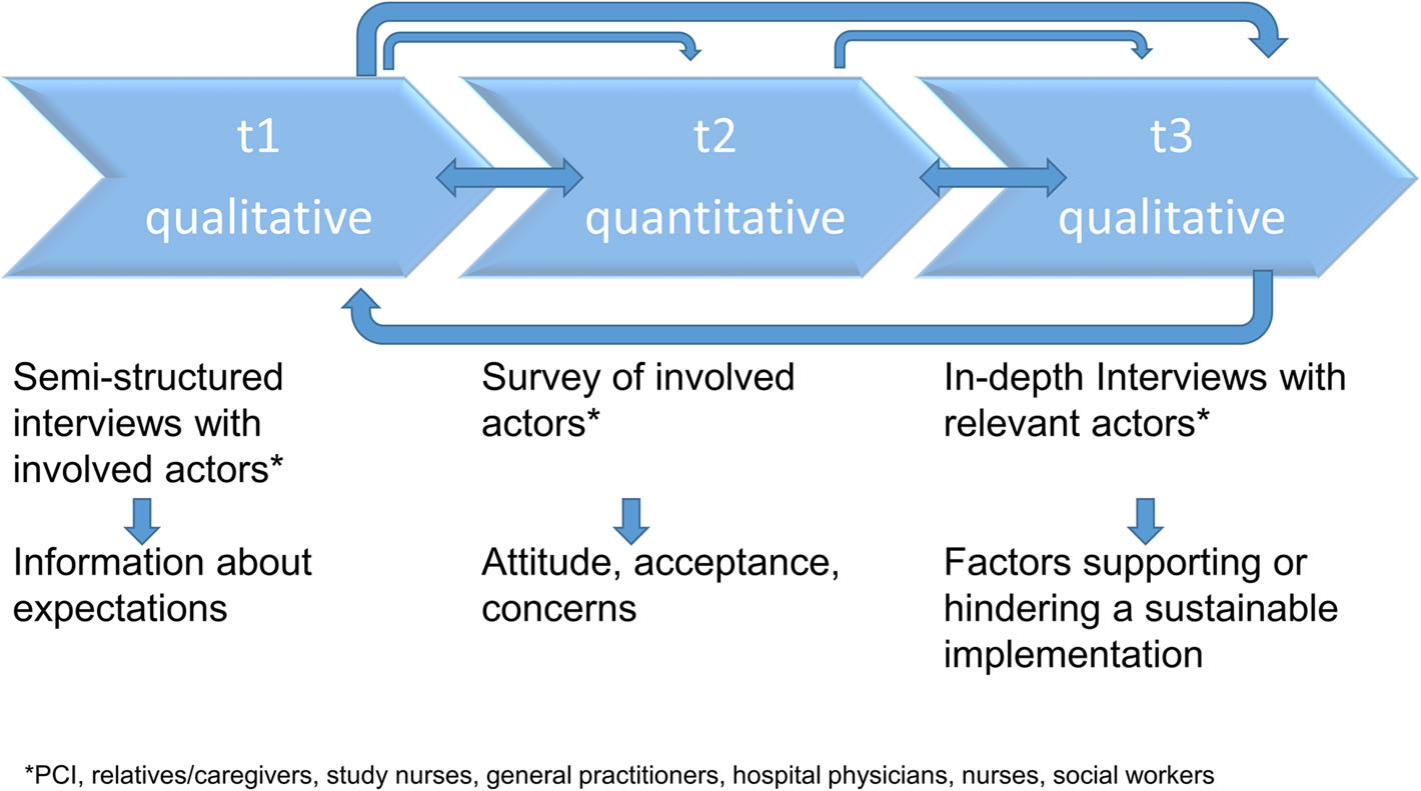
Mixed-methods design in the intersec-CM study process evaluation

The process evaluation of the ICM will focus on the expectations, attitudes, acceptance, concerns and experiences with supporting and hindering aspects of the implementation of discharge management in different groups of actors in routine care. The selection of actors has to be as comprehensive as possible but still manageable for research purposes. Based on the ANT, a mixed-methods design was developed to combine and integrate qualitative and quantitative evaluation methods (see Fig. 2).

At the two study centres Bielefeld and Greifswald, respectively, interviews as well as a questionnaire-based survey with PCI, their caregivers/relatives, study nurses, nurses, hospital physicians, social workers from the respective hospital and general practitioners will be conducted (total 14 per site). This will be done twice, so that a total of 2 × 14 = 28 interviews will be available. Because of the two sites of the trial, we will have 28 × 2 = 56 interviews in total at the end of the process evaluation assessment. With this, we will assess different perspectives of the actors of health care for older PCI in the data analysis. For the survey, all named actors, recruited PCIs and their relatives, the involved stakeholders from the hospitals and the general practitioners, will be included in the questionnaire survey.

The key dimension of the intervention was defined and extended with subdimensions, research questions as well as with the form of data collection and data analysis. Common methods of mixed-methods data collections were integrated, especially interviews and the survey. The qualitative parts include interviews based on an interview guide (phase 1) and later in-depth interviews with narrative modules (phase 3), which will be analysed by using the qualitative content analysis according to Kuckartz [27]. The quantitative survey will be based on standardised questionnaires (phase 2), which will be developed based on the findings that result from phase 1. The results in phase 1 are the basis for the definition of specific questions for phase 2. The results from phase 2 then are the base for the definition of the in-depth questions for phase 3.

### Phase 1: 1 December 2018–31 December 2019—semi-structured interviews (qualitative)

Based on a review of international literature, the first topic guides have been developed with the aim of asking all participants about the basic procedures and experiences with care before the intervention. This involved the time experienced during hospital admission and treatment, as well as discharge from hospital and continued primary care for persons with cognitive impairment. Semi-structured interviews with general practitioners, study nurses, hospital physicians, nurses, social workers, PCI and caregivers have collected qualitative data to determine expectations with respect to the trial as well as to identify supporting and hindering factors for a sustainable implementation of the ICM into routine care.

### Phase 2: 1 January 2020–30 October 2020—evaluation questionnaires (quantitative)

Based on the results of the semi-structured qualitative interviews, a questionnaire has been developed for the subsequent standardised postal survey of all relevant stakeholders of the ICM study who have experienced with the intervention and who are able to assess this. The following have been determined: experience with the project; satisfaction with the intervention, with the qualification of the care managers and with the cooperation with them; challenges of the intervention; and implementation difficulties.

### Phase 3: 1 November 2020–31 May 2021—in-depth interviews (qualitative)

In-depth interviews will be conducted in the third step. Thus, the quantitative questionnaire will be followed by another qualitative data collection based on a convenience sample of all seven groups of actors to broaden the evaluation results of the ICM and to specify supporting and hindering factors for the implementation. Topic guides will be derived from qualitative and quantitative data and subsequently critically discussed within our interdisciplinary research team. Emerging questions will be tested for their relevance and whether or not they are in line with the research questions of the main study. Questions that do not fit the criteria will be eliminated [28].

### Dimensions and research questions

This first draft of this design has been revised with the scientific advisory board of the intersec-CM study in January 2018 including representatives from the German Alzheimer’s Association. Their feedback helped to improve both the main study and the process evaluation (for instance: characteristics to consider when talking to PCI, framework conditions for conducting the interviews such as appropriate length and place as well as ethical considerations and concerns that need to be addressed). We describe the main outcomes referring to the intersec-CM study [see Table 1]. The implementation of the ICM into routine care will be another focus. One part is the satisfaction of the actors, and another part is the feasibility in the healthcare system. In addition, we will analyse how responsibilities will be assigned to actors and describe the clinical pathways in the intervention. Furthermore, context factors are relevant dimensions of the intervention itself. These context factors are, for example, the study region and the hospital structure. In order to determine the differences between the rural area in Greifswald and the urban area of Bielefeld, this factor is taken into account in this process evaluation. Another context factor is a new German legislation—introduction of some aspects of discharge management in German hospitals. Because discharge management is practiced differently in the hospitals, it is important to consider this factor in this study [29].

**Table 1:** Dimensions and research questions assessed in the process evaluation of the intersec-CM study

Dimensions of the intervention	Research questions	Data source
1. Outcomes	Which of the effects on primary and secondary outcomes were statistically significant?	Trial data
2. Implementation/satisfaction	Which expectations and attitudes do patients and stakeholders have with respect to the trial? Which problems occurred during the intervention? Was the intervention delivered as intended to the patients? How is the intervention accepted? Are some intervention components more accepted than others?	Interviews
3. Transfer into routine care/feasibility	In which way is a transfer of the intervention into routine care possible? What are the enablers and barriers to implement the performed Care Management in routine care?	Interviews
4. Assignment of responsibilities and tasks; clinical intervention pathways	How is the intervention conducted? How are responsibilities and tasks distributed and assigned? Which actor fulfils which tasks? Are there typical pathways during the interventions?	Interviews, trial data
5. Qualification and qualification requirements	What qualification is needed to conduct the intervention and to actively support the transition process?	Interviews, questionnaire
6. Context factors	Are there any differences between the two centres? Are there contextual/environmental factors which have the potential to acceptability and/or influence the implementation?	Interviews, questionnaire

### Recruitment

The recruitment of the actors will be closely related to the trial settings. In the hospitals, we will recruit the PCI, their caregivers, the hospital physicians, the nurses and the social workers in the Neurology, Geriatric, Nephrology, Gastroenterology and Trauma Surgery wards. It is important for this purpose that the respondents from the professional fields of work should not only know the processes and the division of labour in the health care system “from theory” or from their training or studies, but also from the corresponding practice that they have experienced in their actual daily work. That is why the inclusion requirement for all the health care professionals is a minimum work experience of 3 years after residency. Furthermore, we will try to interview at least one woman and one man. The nurses have to work in full time (night shift and day shift) to be eligible. With respect to the caregivers, we will try to recruit one young and one older person and one man. In the outpatient setting, we will recruit the general practitioners. The general practitioners should work in a rural and in an urban region of the intersec-CM trial. The study nurses are linking the hospital and the outpatient setting. For our recruitment, one study nurse should work in a rural region and one in the more urban region. The main inclusion criterion for the actors to interview is that a contact with the ICM (as an actual event or as a concept/idea) must have taken place (Table 2).

**Table 2:** Interview partners

Sample	Number	Region rural/urban	Gender f/m	Work experience 3 or more years
PCI	4	2/2	2/2	–
Caregivers	4	2/2	2/2	–
Hospital physicians	4	2 /2	2/2	4
Nurses	4	2/2	2/2	4
Social workers	4	2/2	2/2	4
GP	4	2/2	2/2	4
Study nurses	2	1/1	2/0	2
	26	13/13	14/12	

For all evaluation phases, the study nurses as well as the members of the research team at the two study centres are the gatekeepers. The study nurses will address actors with the stated characteristics and invite to interview appointments. The survey will be conducted both as paper-pencil and online version, respectively, and will be distributed to all involved stakeholders: PCIs and caregivers/relatives will get the survey as paper-pencil and as online version if demanded. The same alternatives will be provided to the general practitioners. The hospital staff (physicians, nurses, social workers) in the involved wards will receive the survey via their occupational e-mail addresses. In case of low participation, the study nurses will personally distribute the surveys on the wards. No recruitment step will include incentives. A written informed consent will be solicited from each participant.

The process evaluation covers a total of 3 years and starts in the seventh study month. The first data collection, processing and analysis has taken place at the end of 2018 and whole of 2019. The identification of the expectations and the potential positive effects are scheduled for the first and second quarter of 2020. The quantitative survey (including preparation, data collection and analysis) in the seven target groups takes place in the last two quarters of 2020. The concluding qualitative in-depth interviews for the identification of potentially supporting and hindering factors of a sustainable implementation of ICM are planned at the end of the fourth quarter of 2020 until 31 May 2021.

### Interview procedures

The selection of interview partners will be conducted according to a purposeful sampling approach with an emphasis on variation of actors’ insights in the implementation process [30]. Patients will be interviewed at least 3–4 weeks after discharge from the hospital, if required in the presence of relatives at home. Interviews are conducted in accordance with the criteria proposed by the American Bar Association Commission on Law and Aging & American Psychological Association (2008) for older adults with diminished capacity [31]. These include, among others, medical and psychiatric diagnoses that may contribute to diminished capacity. With this knowledge, the risks associated with the specific person and situation are identified before—and during the interview. The person’s decision-making ability and cognitive function as well as psychological and emotional factors are observed and action is taken accordingly: The interview will be paused or, if the interviewee is emotionally affected, the interview will be terminated and professional support will be offered. Conducting an interview with a PCI can be challenging for various reasons. Therefore, interviewers were specifically trained to pay attention especially to PCI’s body language and the establishing of a reliable and trustworthy relationship with the participant. Further, interviewers were made aware that interviews should be conducted at a relaxed pace—allowing pauses and breaks for the PCIs to gather their thoughts and to prepare their answers internally.

The quantitative data will be collected from a maximum of all relevant actors, i.e. the questionnaires will be widely distributed. So, all patients and their relatives randomised to the intervention group (approx. *n* = 300) will receive the survey. Also, all GP (approx. *n* = 90) of these patients, as well as all physicians (approx. *n* = 50), social workers (approx. 20) and nurses (approx. *n* = 80) participating wards of the two hospitals will be asked to complete the surveys.

### Data collection and analysis

The data of the interviews in phase 1 and phase 3 will be audio recorded, verbatim transcribed according to Dresing and Pehl [32] and analysed by the qualitative content analysis as suggested by Kuckartz using the software MAXQDA [27]. The coding of the interviews will be conducted by two members of the study team in the consensual coding approach. In a first step, both team members code the interviews separately. In a second step, both coders compare their respective category systems with respect to similarities and differences. Differences will be discussed, and the category systems will be modified if both coders agree. In the majority of cases, this will cause an extension of the category system. Subsequently, a system with categories, sub-categories and codes will be developed based on the code systems of both coders. The results of the qualitative interviews in phase 1 will be the base for the standardised questionnaire in phase 2. Finally, the results of the qualitative interviews will be compared with the results of the questionnaire to answer the research questions.

Quantitative data will be analysed by summary statistics like mean, median, minimum and maximum for continuous variables, and frequencies and percentages for categorical data will be calculated. To identify differences between the different actors and regions, Fisher’s exact test will be conducted.

The transcripts of the interviews will be blinded by pseudonymisation for data analysis. Therefore, personal data about the interviewed subjects will not be reconstructable by people who were not involved in the research project. The questionnaires to the stakeholders will be anonymous.

Every major step of the collection and analysis of data in the evaluation process will be reflected by the research group. This reflection will be documented with memos throughout the whole research process based on Grounded Theory [27].

## Discussion

We have presented the dimensions, research questions and methods of the process evaluation within the intersec-CM study. On the one hand, “mixed methods” in this process evaluation means that the three phases of our assessment will build on each other. On the other hand, it means that we sometimes will ask the same questions at different times to investigate barriers and promoting contents of the ICM for older PCI during and after hospital stays. In this way, it will be possible to validate the results of one method by using another method and thus obtain more meaningful and reliable results. However, the main aim of process evaluation will be the documentation and analysis of the intervention.

Challenges in data collection will include the development of an interview guide which requires different terminologies depending on every specific actor in the intervention. Conducting these interviews, there is a risk of focusing on issues that are interpreted differently by the interviewers than by the actors themselves. In addition, the interviews with older PCI are expected to be difficult to conduct [32]. However, the use of qualitative and quantitative approaches as different techniques of process evaluation (triangulation of the findings) may help to capture and analyse the data on different dimensions of the intervention.

## Conclusion

We present an approach to evaluate the process of a complex intervention in health care of older PCI in the intervention arm of a randomised controlled trial. The design may serve as a stimulus for process evaluations of similarly designed complex intervention studies. We integrated current publications on process evaluation/evaluation design in complex trials and a number of recommended methods to investigate the complex structure of an intervention at the interface between inpatient and outpatient care. We have described dimensions and the suitable methods to assess relevant data. Contribution can be made to further methodological development of process evaluations and to identify suitable analytical instruments for future evaluation research. The results can also be used to apply the process evaluation for intersectoral care management concepts to other clinical issues or patient groups.

If the RCT will lead to a positive outcome, we will be able to identify enablers and barriers for implementation of the ICM into routine care. Based on this, a checklist based on the main results of the process evaluation will be created to prepare an implementation guideline for routine care in the German healthcare system. This checklist might supply some answers for a better transition management for older PCI in Germany and to inform recommendations for the German guidelines on medication, medical aids, therapies and ambulatory care [33]. In addition, the findings of this process evaluation will generate new knowledge for the development of interventions that intend to improve dementia care management.

### Process evaluations status

The process evaluation is at the stage of phase 1 (semi-structured interviews/qualitative), first interview: 6 December 2018. The approximate date when recruitment to the process evaluation of the study will be completed is 31 May 2021.

## Data Availability

There is no plan to provide public access to the data.

## Abbreviations

ANT: Actor-network theory
CAU: Care as usual
DelpHi-MV: Dementia: life- and person-centred help in Mecklenburg-Western Pomerania
GP: General physician/general practitioner
ICM: Intersectoral care management
Intersec-CM: Intersectoral care management for older people with cognitive impairment during and after hospital stays
PCI: People with cognitive impairment
RCT: Randomised controlled trial
